# “*How Can I Gain Skills if I Don’t Practice?*” The Dynamics of Prohibitive Silence against Pre-Marital Pregnancy and Sex in Zimbabwe

**DOI:** 10.1371/journal.pone.0053058

**Published:** 2013-01-23

**Authors:** Jeremiah Chikovore, Lennarth Nystrom, Gunilla Lindmark, Beth Maina Ahlberg

**Affiliations:** 1 HIV/AIDS, Sexually Transmitted Infections, and Tuberculosis Unit, Human Sciences Research Council, Durban, South Africa; 2 Malawi Liverpool Wellcome Trust Research Programme, Blantyre, Malawi; 3 Division of Epidemiology and Public Health Sciences, Department of Public Health and Clinical Medicine, Umeå University, Umeå, Sweden; 4 Section for International Maternal and Child Health, Department of Women's and Children's Health, University Hospital, Uppsala, Sweden; 5 Department of Women’s and Children’s Health, Faculty of Medicine, Uppsala University, Uppsala, Sweden; 6 Skaraborg Institute for Research and Development, Skovde, Sweden; Vanderbilt University, United States of America

## Abstract

Young people face sexual and reproductive health (SRH) problems including Human immunodeficiency virus (HIV) and Acquired immunodeficiency syndrome (AIDS). It is critical to continue documenting their situation including the contexts they live in. As part of a larger study that explored perspectives of men to SRH and more specifically abortion and contraceptive use, 546 pupils (51% female; age range 9–25 years) from a rural area in Zimbabwe were invited to write anonymously questions about growing up or other questions they could not ask adults for fear or shame. The pupils were included following descriptions by adults of the violence that is unleashed on unmarried young people who engaged in sex, used contraceptives, or simply suggested doing so. The questions by the young people pointed to living in a context of prohibitive silence; their sexuality was silenced and denied. As a consequence they had poor knowledge and their fears and internal conflicts around sexuality and pregnancy were not addressed. Current action suggests concerted effort at the policy level to deal with young people’s SRH in Zimbabwe. It nevertheless remains necessary, as a way to provide support to these efforts, to continue examining what lessons can be drawn from the past, and how the past continues to reflect in and shape present dynamics and relations. There is also need to look more critically at life skill education, which has previously been described as having failed to address adequately the practical needs of young people. Life skill education in Zimbabwe has rarely been systematically evaluated. A fuller understanding is also needed of the different factors co-existing in contemporary African societies and how they have been and continue to be constituted within history, and the implications to the promotion of adolescent SRH.

## Introduction

Young people continue to face sexual and reproductive health (SRH) problems including Human immunodeficiency virus (HIV) and Acquired immunodeficiency syndrome (AIDS [1]. Five million young people aged 15–24 are HIV infected, with an estimated 900 000 new HIV infections having occurred in this group in 2008 alone [2]. Over half of the 340 million new sexually transmitted infections (STIs) other than HIV occurring annually are among young people aged 15–24 [2]. High rates of pregnancy and unsafe abortion are also reported for the group [3]–[6] – in Zimbabwe, the teenage pregnancy rate is 21% for age 15–19 and adolescent fertility 120 per 1000 girls and 70 per 1000 girls for rural and urban areas respectively [7].

The past decade-and-half has witnessed a shift within SRH research and policy from an almost exclusive focus on adults, particularly married women – which was premised on biomedical notions [8] and thus ignored how young people are sexually active beings who require to be engaged before they reach childbearing age [4], [9] – towards recognising young people as a priority group for health promotion [10], [11], [12]. The growing interest in young people is driven largely by a concern with poor health outcomes [10] and evidence suggests this shift in focus may have started to generate results: the recent decline in HIV prevalence in high burden African countries including Zimbabwe has, for example, been partly attributed to behaviour change among young people [2], [13]. Zimbabwe is among the first countries worldwide to implement a National Behavior Change Strategy with an explicit focus on making SRH information available to adolescents [14]. The country is also among high HIV burden countries that have successfully reduced prevalence among young people by at least 25% [2]. Young people now wait longer to become sexually active, have fewer multiple partners, and those with multiple partners have increased their use of condoms [2].

Yet, still relatively poor, adolescent SRH demonstrates a persisting failure by young people to access services and information [15]. In addition, despite the complex relationship between knowledge and attitudes, and behaviour change [16], research on young people has frequently used quantitative survey-based analyses [17]. To support the growing focus on adolescents and the improvement in health outcomes being witnessed, it is necessary to adopt context-based analyses that simultaneously draw lessons from the past and more specifically examine the role of past and on-going processes in shaping contemporary social relations. In this paper, we use self-generated questions written by young people to reflect on the role and historical constitution of some actors in contemporary Zimbabwe in adolescent SRH.

In pre-colonial African societies, for example, there were structures for managing transition from childhood to adulthood, regulating sexuality and reproduction [18] and guaranteeing economic security for women. Colonial interventions including taxation and land alienation impoverished indigenous African people and reduced women’s control over produce from land [19]. This resulted in labour migration that separated husbands and wives. One of the outcomes was emergence of commercial sex work by single or widowed women around white-owned enterprises [19], which further altered gender, family and community relations, distorted socialization structures and transformed the meaning and practice of pre-marital sex. Schooling and labour migration continue to separate family members for varying lengths of time [20]. This, and the fact that many parents may not have the skill to handle their children’s developmental needs, limits contemporary nuclear families’ ability to play a positive role in adolescent SRH.

The situation in Zimbabwe at the turn of the century illustrates a common pattern for many modern-day African societies, where multiple actors with diverging views on adolescent SRH exist side-by-side. According to the country’s National HIV/AIDS Policy [21], sexually active young people including those below 16 years were allowed to receive counselling and SRH services. Yet, a girl under 16 years was, under another law, not mature enough to consent and having sex with her was thus considered to constitute statutory rape. Health workers were found to report girls infected with STI to the police as suspected cases of sexual abuse [22].

The clash among actors seemed to affect the imparting of life skill and AIDS education. Even though the AIDS education program that was being implemented did not mention condoms, some parents, churches and teachers opposed the programme as being too explicit and advanced for primary school pupils [23]. The Ministry of Education also refused to grant some UNICEF researchers permission to interview pupils less than 16 years on sexuality issues, arguing ‘this might make them sexual’ [24].

## Methods

### Design and Setting

This study was conducted within a larger qualitative study exploring male perspectives on abortion [25]. The men in the larger study described the violence unleashed in the family when a girl became pregnant, engaged in sex or used contraceptives before marriage. This study thus focused on young people to explore their experiences living within the context described by the men.

The study was carried out in a ward in Chiredzi District, located in Masvingo, a predominantly rural province in south-eastern Zimbabwe. The district comprised of rural village settlements, sugar estates, and wildlife conservation parks and had an estimated population of a quarter of a million. At the time of study, in 2001, male labour migration to the plantations and to South Africa led to long periods of spousal and family separation. The women assumed a heavy burden of looking after families, with little support from their migrant husbands, some of who spent long spells of time without communicating. It emerged that some women felt abandoned and resorted to casual labour or selling sex for family and personal survival. Women said they felt justified to use contraceptives to prevent or limit childbearing while men, on the contrary, viewed contraceptive use as enabling married women to conceal evidence of extramarital sex when husbands were away. We have reported elsewhere how these opposing views led to male violence around contraceptive use [25].

We also report elsewhere [26] how initiation rituals for both adolescent boys and girls were still practised in the study area, albeit in ways that illuminate the range of actors but more specifically the conflict that ensued between the schooling system and the community. Parents felt that the schooling system was promoting condom use by teaching about sexuality, while the schooling system felt parents and the communities, in particular the initiation ceremony, interfered with the schooling system, and described them as being retrogressive [26]. These dynamics are characteristic of societies that are marked by what has been termed ‘hybridity’ [27], [28], which involves multiple actors with different interests co-existing and interacting within specific local contexts, while at the same time responding to wider external forces. Some of the actors embrace change and yet others resist it, and occasionally there is visible clash of values. In this sense, concerns expressed by parents regarding the impact of schooling on adolescent SRH can be seen as one way of countering the perceived encroachment of external influences onto what they consider to be their cultural group’s identity and values. Because they are considered ‘beings in the making’ [29], young people are also seen as prone to embracing change easily and indiscriminately. Parents and communities thus take it upon themselves to regulate the content of sexuality teaching in schools as a way to fortify against cultural encroachment. In the process they simultaneously tout the supposedly superior value of their own traditional mechanisms of socialization.

### Data Collection

Data was collected in 2001 from 546 pupils (51% female) attending one secondary and two primary schools in the same area where earlier studies had been undertaken. Apart from one pupil who was aged 25, the rest were aged 9–18. A self-generated question method, previously used by Ahlberg and colleagues [30] was chosen to allow pupils to express themselves more freely and to avoid shortcomings reported in literature regarding interviewing young people about sensitive matters of sexuality [31]. Pupils were invited to ask questions on issues of growing up, or those questions they could not ask their parents, teachers or other adults for fear or shame. It was stressed that the pupils must write the questions anonymously, indicating only age, sex and class level, and use the language in which they felt most competent to express themselves. The researchers emphasized that they sought to learn rather than to teach the pupils. Moreover, pupils were told that the information would be brought to the attention of authorities to help better address concerns of young people. The pupils generated over 3000 questions (for details of the procedure, see [26]).

#### Ethics

Ethics approval for the study was granted by the Medical Research Council of Zimbabwe and the Research Ethics Committee of the Medical Faculty, Uppsala University, Sweden. Further permission for the study was sought from and granted by the local chief, provincial and district administrative and educational authorities, and school heads and teachers of the specific schools that were included. The school heads and teachers were informed of the study goals, and the anonymous procedure for gathering data from the youth. Pupils were informed about their right to not participate, or to withdraw without compromising their schooling. They were also informed that the data would be used anonymously and, except for the researchers, neither teachers nor any other adults would have access to the raw data. Although pupils were briefed in the presence of their teachers, they were also informed that the teachers would leave the room, or would not move about while the pupils wrote. Certain considerations made it practically difficult to obtain consent directly from parents or guardians in this rural context. Firstly, rural homes in these villages are sparsely located, and were difficult to access owing to the terrain, particularly during wet season. Visiting homes would also require that the research team was accompanied by the children individually, which could not be done without significantly disrupting schooling. Given the nature of the exercise and the challenges of contacting parents, and also precautions that were taken, it was deemed that teachers and school heads acting in ‘loco parentis’ would suffice in this instance. Pupils took part actively and animatedly, indicating they were excited at the opportunity to write and ask issues that they otherwise had no possibility to inquire about from adults. The entire exercise lasted 20–30 minutes for each class that was visited to minimise disruption to the curriculum.

### Data Analysis

The self-generated questions were transcribed, and statements written in local language were translated into English. Those originally in English were retained with only minor editing for grammar. The text was content analysed with a focus initially on organizing the large volume of data through grouping questions that belonged together. This descriptive coding led to nine preliminary categories, namely HIV/AIDS, pregnancy and reproduction, sexuality, maturation, STIs and genitourinary health, abortion, contraceptive use, marriage, and education. ‘HIV/AIDS’ was the most frequently mentioned topic (75% of all pupils); 54% cent mentioned pregnancy, 49% sexuality, 21% maturation, 20% STI and genitourinary issues, 17% abortion, 17% contraceptive use, and 11% marriage. More analytical coding involved reading the questions against the study goals while at the same time identifying new unanticipated themes. Sexuality emerged as the core category [32] which ran through the rest of the issues, including HIV and AIDS. However, because most question on HIV and AIDS pertained specifically to this theme rather than the wider conduct of sexuality, the theme of HIV/AIDS has been reported separately [26]. The data were merged, split, and re-connected in theoretical ways to generate consolidated themes built around dimensions of the core category, until no new themes or dimensions could be identified, or saturation had been achieved. We present the themes illustrated with age- and sex-linked quotes.

## Results

### A. Youth Sexuality in Contexts of Prohibitive Silence

The self-generated questions suggested young people were curious about sexual matters. The younger ones inquired why adult men and women slept in secluded spaces. While the curiosity seemed related to young people’s development and the sexual feelings that accompany the maturation processes, it also suggested attempts by the young people to make sense of a paradox where sexual mixing is not permitted for them but adult men and women openly sleep together:

*‘Why do they say children mustn’t enter where their mother’s sleeping with their father?’ (girl-10)*

*‘What time at night do they have sex, exactly what time?’ (girl-11)*

*‘What does it mean when a woman shares the same blanket with a man?’ (girl-13)*

For the older youth, the curiosity shifted from what adults do while in private to how sex is actually performed, and the nature of the pleasure or pain that it generates:

*‘When I have sex with my boyfriend, isn’t it painful?’ (girl-15)*

*‘When people have sex, where exactly is the nice taste located?’ (boy-16)*

*‘I want to know how deep the penis goes.’ (boy-15)*

*‘What type of friction do a penis and a vagina produce?’ (boy-19)*

Although the school youth expressed curiosity, they rarely mentioned sex directly in their questions. Occasionally, and illustrating the rhetorical writing and the excitement for being able to write on a taboo subject within a formal setting, sexual organs were mentioned directly by name. Often, though, euphemistic terms were resorted to, such as in the case of the sex act “*zvinonyadzisa*” (meaning embarrassing, disgusting, or socially proscribed). This is significant given one reason for asking these young people to write questions privately and anonymously was to enable free expression. The failure by many to directly write about sex suggests possible internalization of the discourse that portrays sex outside marriage as immoral, or merely lack of terms for describing the sexual act.

The curiosity about sex on the one hand, and moralistic discourses on the other, led to internal conflict which in turn affected how the youth exercised their sexuality. The youth especially girls were, for example, ambivalent and uncertain regarding how they should behave in sexual situations, or even who should initiate the sexual act:

*‘When I want to have sex, what do I do? Is the boy the first one to ask? Or maybe I’m the one to say, Why don’t we do what the adults do? I really want to know.’ (girl-15)*

*‘What am I supposed to do during sex with my boyfriend?’ (girl-15).*

Unlike girls who were ambivalent, boys expressed keenness to have sex. They described sex as the ultimate sign of love, in addition to being necessary for developing their sexual skills:

*‘Are we allowed to practice having sexual intercourse so it becomes easier when we get married?’ (boy-17)*

Curiosity about sex – which is socially sanctioned – and a parallel desire to be morally upright added to the internal conflict. The latter is visible in statements such as the one below, where a girl excuses herself for being in love but at the same time blames herself for giving in to a boy:

*‘When a boy and a girl are in love, who lies down first? But, really, to end up allowing a boy to touch your breasts, what’s really happened?’ (girl-14)*

### B. Peer Pressure to have Sex

Across the age range, boys felt compelled to have sexual intercourse preferably with multiple partners, partly believing this improved their sexual skills. They thus wondered why the girls were not always forthcoming:

*‘Why do they say you should “nyenga nyenga” (sex all over the place)?’ (boy-13)*

*‘I’m in love with a girl aged 15 but she refuses to have sex with me, is that good? Sex is the sign of love so it must be allowed. How can I gain more skills if I don’t practice?’ (boy-18)*

The divergent expectations between boys and girls seemed to affect their interaction. It was clear, for instance, that there was poor communication, and boys were inclined to use violence against girls as a result. In turn, the girls, who were poorly equipped or prepared to deal with the violence, struggled to make sense of how violence could occur in what are otherwise expected to be love relationships:

*‘What am I supposed to do if a boy forces me to love him when I don’t want to? What if a girl doesn’t want sex but the boy forces her, even though they’re in love?’ (girl-17)*

The pressure to be sexually active was not limited to the boys. Rather, both boys and girls coerced peers into becoming sexually active by making anxiety-raising claims about the fate that awaited those not sexually active. That the girls also circulated this type of information despite the ambivalence observed earlier suggests how complex young people’s SRH world is. One claim was that pimples on the face were caused by either excess or lack of sexual intercourse. Portraying normal and inevitable stages of growth and development as potentially pathological was bound to generate significant distress, as the following statements indicate:

*‘I don’t want to have sex until I marry my own wife… I have pimples on my face, and my friends say it’s because I don’t practice sex with girls, is that true? To me, this is a hard saying. At my age I don’t want sex and I don’t want these pimples. How can I get rid of these harsh sayings?’ (boy-17)*

*‘I want to know how teenagers can get rid of pimples on their faces. Once I asked other people. They said that to get rid of them I had to have sex as many times as possible.’ (boy-18)*

Complications allegedly arising from abstaining from sex were circulated among girls as well:

*‘When a boy stops me, I shudder. When I tell other girls my age that I haven’t had sex yet, they say that on that day I’ll see fire. Why should I see fire?’ (girl-15)*

*‘I once was told by my friend that if I refuse sex my hole won’t be opened and I’ll have problems when I give birth. Is this true?’ (girl-16)*

### C. Fear of Pregnancy

A major aim in including the young people in the broader study was to explore further the views expressed by adults: that teenage pregnancy promoted violence at the family level against the girl or her mother. The young people confirmed they were anxious about what might happen in the event of a pregnancy. Girls specifically mentioned the threat of being thrown out of home or suffering some other undefined but grave consequences. The anxieties were especially burdensome because the youth were unable to talk about pregnancy with parents or friends:

*‘If I’m pregnant but I’m afraid to tell my mother or friends, what should I do?’ (girl-12)*

*‘If you’re a young girl and you get pregnant, what’ll be done to you?’ (girl-13)*

*‘If I get pregnant before I finish school and my parents throw me out, what should I do?’ (girl-16)*

However, several contradictions emerged in the ways boys viewed premarital pregnancy. For one, having indicated they put pressure on girls to have sex with them, boys also expressed fear about being forced to marry a pregnant girl when not entirely certain about paternity. They were additionally concerned about taking on financial responsibilities for a family before they had become financially secure. Termination of schooling appeared equally worrisome to the boys:

*‘What if I make a girl pregnant and I’ve no money?’ (boy-12)*

*‘What should I do if I’ve made a girl pregnant and she used to have many boyfriends and they want me to marry that girl?’ (boy-16)*

*‘What’s wrong with a person continuing their education after they get married? If a person marries, will that disturb their schooling?’ (boy-18)*

*‘If I make a girl pregnant while I’m still in school, can I still go to school to prepare for my life?’ (boy-17)*

The boys also worried about having to face the girl’s parents, or experiencing violence from their own parents. At the same time, they feared the embarrassment of facing their peers in school after making a girl pregnant:

*‘My problem is when I tell my parents about my girlfriend’s pregnancy, they beat me. What can I do if I have a problem like that?’ (boy-17)*

*‘My problem is that when I had sex with my girlfriend she got pregnant. I don’t know how old the pregnancy is and I’m afraid to tell my parents. My head is aching because of this.’ (boy-17)*

*‘We drop out of school because we have sex in the belief that we’re playing, but then she gets pregnant. Her parents confront me, and for me to continue with school, I’d be too embarrassed to face my friends who’d laugh at me.’ (boy-15)*

Moreover, despite putting pressure on girls to have sex in the first place, the boys blamed girls who became pregnant:

*‘And what is a girl really looking for when she gets pregnant while she’s young and the pregnancy isn’t acknowledged by the man? He denies and says it’s not his, and the girl gets stuck at home?’ (boy-13)*

In a way that illuminates how younger boys related to older men within their communities, boys castigated the latter for enticing young girls using wealth. At the same time, the need for girls to keep their sexuality under check, avoiding men and hence problems related to pregnancy was stressed by younger pupils of both sexes:

*‘Whether you’re old or young you should control yourself… If you’re a girl you may stop going to school because of this love for “varume” (men), but then you get rejected and you end up suffering.’ (boy-13)*

*‘If you’re a woman and you leave your home and go to a man’s place, what are you really looking for?’ (girl-13)*

*‘A schoolgirl drops out of school because of excessive love for boys. We schoolchildren can’t control ourselves well enough to stay in school.’ (girl-13)*

*‘Why, really, does a girl get to be a “hure” (prostitute)? If she gets pregnant before she’s married, then what happens to her? Those who don’t listen suffer the consequences.’ (girl, age missing)*

Girls in turn described the ensuing tensions around pregnancy that then compelled them to consider abortion, itself problematic due to the moral implications, and fears of physical, social, and legal consequences. The fact that they could not communicate with other people, even their parents complicated their situation even further:

*‘If I have an abortion, won’t people say I’m a fool? If you get pregnant by a boy “musango” (in the bush/outside marriage) and you want to remove it, what do you do? Won’t something go wrong as you remove?’ (girl-12)*

*‘We see girls who have removed pregnancy; some bleed and die.’ (girl-12)*

*‘If you remove pregnancy, and your parents hear about it, won’t they throw you out?’ (girl-13)*

*‘Suppose a person gets pregnant and removes the pregnancy, will they get arrested?’ (girl-13)*

*‘What are the methods of abortion when my parents say they want to kill me?’ (girl-15)*

*‘If I’m pregnant and I want an abortion, how should I ask my mother?’ (girl-16)*

For the boys, tensions emanating from making a girl pregnant or simply being acussed also forced them to consider migrating as an escape strategy The challenges of living under difficult and unfamiliar circumstances after migrating were still considered as being more tolerable than staying to face the consequences of having a pregnant girlfriend. In addition, as they considered various options to deal with a pregnant girlfriend, thoughts of helping the girl secure abortion also entered the boys’ minds:

*‘I can’t think of ways to prevent pregnancy. We don’t have anyone to teach us and give us books on these issues. For instance, if she gets pregnant, what do you do?’ (boy-17)*

*‘How is abortion done when my girlfriend is pregnant?’ (boy-17)*

### D. Protest and Use of Substitutes to Prevent Disease in Contexts of Denial of Service and Information

Protests directed against the denial of services and information to young people and the moralization of sexuality thus formed a recurrent theme in the questions:

*‘What’s wrong with a relationship between a boy and a girl?’ (girl-16)*

*‘Once my parents know that you’ve a boyfriend or girlfriend, they throw you out, even if you’re 18.’ (boy-17)*

The youth described how they resorted to substituting “*freezit*” (a polythene refreshment container shaped like a male condom) for the hard-to-obtain condoms, or simply reusing previously used condoms just to prevent being infected with HIV. In the absence of preventive services, they implied they are also forced to make the difficult choice whether to have sex, enjoy, and suffer the serious consequences including death, or to abstain and forego the sexual pleasure:

*‘Is it okay to pick a used condom and use it?’ (boy-14)*

*‘How can we avoid being attacked by diseases that you get when you have sex?’ (boy-17)*

*‘If I sleep with a girl using a “freezit” do I get an STD or AIDS?’ (boy-18)*

Given the muting of adolescent sexuality, young people would also not disclose or seek treatment when they contracted STI:

*‘I had an affair with a girl I didn’t trust. When I slept with her, I developed sores on my anus that itched. I didn’t tell anybody. I was ashamed to tell my parents. I was afraid to go to the clinic or to face my friends. But if I don’t sleep with her, they heal, when I sleep with her, they reappear.’ (boy-20)*

Finally, the questions also indicated the poor knowledge young people had on sexual issues, including contraceptives and maturation processes:

*‘I say children shouldn’t be given pills because if they use them, pills will damage their womb and in the end they won’t be able to have children or they’ll get sick.’ (girl-15)*

*‘The problem is that I’m having wet dreams. I told my friends and they told me it’s a sign I won’t have children or be able to make a girl pregnant. Is that true?’ (boy-18)*

*‘I hear others say that condoms have “tumakonye” (little worms/germs) inside them that you can see if you put a drop of water in the condom. Please tell us the truth because now we’re afraid of using them, which is causing pregnancy and disease among schoolchildren.’ (boy-25)*

## Discussion and Conclusion

Growing concerns with young people’s vulnerability to HIV/AIDS have seen adolescent SRH being made a part of the national agenda in several countries [11]. In terms of research, however, cross-sectional designs – which permit little room to assess causality – have been largely relied on [17]. As well, contextual issues are rarely a major focus whether in ‘government policy, the economic climate, family functioning, school climate and relationships, peer or community; rather, most research focuses on adolescent knowledge, attitudes, and beliefs with little attention as to how these are derived’ [17]. Young peoples’ understandings of sexual issues have also barely been sought [33].

The data presented here were obtained within an iterative research process [34] where young people in school were requested to ask questions that they could not ask adults or their friends because of fear or shame. They asked the questions by writing them anonymously and confidentially. Excitement at being asked to write on a taboo subject within a formal class setting where open talk about sexuality seldom occurs may have led to some rhetorical questions being asked. At the same time, this opportunity presented to the young people generated insight into their practices and perspectives in sexuality. The data portray poor knowledge and communication; peer pressure, inner turmoil, conflicts and fears around sexuality; frustration arising from failure to access services; lack of skills to negotiate sexual relationships; and general fear around pregnancy and other sequel of sexual activity such as unsafe abortion.

Two issues are important to focus on. The first relates to the social, political and cultural context within which young people live and acquire their senses of gender identity and understandings about sexuality, and which determines the nature of access that they have to SRH services. The prohibitive context, where adults and other actors seek to regulate young people’s sexuality, is increasingly discussed globally and in Africa [35], [36]. Studies out of Africa also frequently demonstrate parents, teachers, politicians, religious leaders, and health care workers denying service and information to youth [3], [22], [31], [37], [38], [39]. In this respect, some researchers consider adolescent sexuality and SRH as outcomes of a combination of historical processes and the mix of actors that have emerged therein [40], [41], [42]. Our data similarly highlight the different actors involved directly and indirectly in adolescent SRH – care workers, policy makers, parents, the educational system, traditional authorities and institutions, and peers among many – and their effects, separately or in interaction, on the young people’s SRH.

While the term ‘hybrid society’ attracts various usages, our use denotes having different actors who may hold different values and perspectives co-existing and interacting in one locale, and also old (traditional) and new (modern) aspects of life mixing and continuing to generate new ways of seeing and doing [27], [28], [43]. This then implies that efforts to improve adolescent SRH require that actors relevant to young people’ sexuality and SRH in contemporary society are identified and mapped through asking: who are these actors? how are they constituted? how do they relate vis-à-vis one another? what value frameworks guide them? and, how have they been shaped by and responded to socio-economic change? Ideally, such mapping ought to also lead to critical reflection even of the actors themselves about their role in adolescent SRH. Similarly, just how knowledge about young people and children generally is generated and made sense of needs to be examined more closely. Shanahan [36] has argued that children were left out in the literature until only recently, following which the focus on the children has been dominated by issues of deviance and delinquency, particularly to illuminate them. Extending a similar argument to adolescents, one notes that this phase of life is frequently cast as a period of upheaval [36] and incompleteness, requiring protection and guidance [29].

The second issue relates more specifically to how the context affects sex and life skill education for young people. Notwithstanding the scant evidence of evaluation of school-based programs [44], sex education has been criticized for failing to link to what the young people see and want in their lives [35]. It is argued that as it attempts to cater for different interests and sometimes conflicting values, life skill education may have become too general, seeking to foster too diverse and complex skills [40]. In general, life skill education faces a myriad of challenges: teachers are reported to be unsure how to teach the subject; they are also embarrassed by it, or tend to emphasize only the negative consequences of sexuality [24], [45]. The participatory learning encouraged in life skill education similarly conflicts with the didactic approach teachers are mostly trained in [40], and puts strain on teachers already burdened with full curricula [9], [45]. In some cases, teachers find themselves juggling the roles of parent and teacher in ways that contradict each other [45]. Some teachers may also be keen to talk about sexuality, but others consider pupils who participate in such classes as being naughty [38].

The youth in this study were largely unaware how to deal with the many issues of sexuality that they raised. In cases where they had some knowledge, such knowledge was incomplete or contradictory. Moreover, they seemed to feel the burden of having knowledge that they could not use to enhance their health or simply to protect themselves. This study thus restates the importance for education to help the youth better manage the challenges that they encounter at the micro-levels of their sexual and peer relationships. This requires, as Rachel Gurevitz [46] states, that education moves beyond imparting scientific understandings. It is worth noting, though, that as debates around AIDS education shift towards syllabi content and the contexts of delivery [45], policy in countries such as Zimbabwe (see for example, [14]) appears to remain at the general level, saying little to address the practical issues this and other studies continue to highlight.

As this study was carried out at the turn of the century, it is necessary to also reflect on the implications of the findings close to a decade later. Since the turn of the century, many African countries including Zimbabwe have experienced a decline in HIV prevalence, partly due to young people waiting longer to become sexually active, having fewer multiple partners, and those with multiple partners increasing their use of condoms [2]. Whereas the decline in HIV prevalence is a welcome and significant development towards realising improved SRH for young people, adolescent SRH, though intertwined with, tends to be much broader in scope than HIV and AIDS. Thus, despite the improvement in their HIV/AIDS indicators, most young people still have limited access to sexual health advice, contraceptives, and HIV counselling and testing services that are appropriate for them [1], [47], [48]. Moreover, life skill education is the single most widespread strategy implemented to deal with adolescent SRH [11]. Along with other strategies such as training staff to meet the needs of young people and respect privacy and confidentiality, having peer educators available to young people; having separate space, time and location that is convenient and affordable and wide ranging services [39], life skill education is rarely universally implemented nor systematically evaluated. In countries with generalized epidemics, fewer than 70% have implemented school-based HIV education in most or all districts [47]. Young people therefore remain vulnerable because they lack access to age-appropriate information through schools, the media, or other sources. Families, communities, together with laws and policies still oppose or fail to support young people in need of services [1] and frequently, adolescent SRH encounters funding and ideological restrictions [39].

In conclusion, efforts to improve adolescent SRH ought to be holistic, inclusive and comprehensive, with multifaceted combination activities at different levels of action [15]. There is need for greater dialogue but also self-reflection among social and political actors. Similarly, efforts aimed at young people, including education, must be assessed not merely on the basis of whether they are being implemented or funded, but also their effectiveness in helping the youth access services, and enhancing their ability to communicate on sexual issues within their relationships and settings.
